# Antibiotic exposure during pregnancy increases risk for childhood atopic diseases: a nationwide cohort study

**DOI:** 10.1186/s40001-024-01793-9

**Published:** 2024-03-20

**Authors:** Sheng-Kang Tai, Yi-Hsuan Lin, Ching-Heng Lin, Ming-Chih Lin

**Affiliations:** 1 Department of Pediatrics, Show Chwan Memorial Hospital, Changhua, Taiwan; 2 Children’s Medical Center, Taichung Veterans General Hospital, Taichung, Taiwan; 3 Department of Post‐Baccalaureate Medicine, College of Medicine, National Chung Hsing University, Taichung, Taiwan; 4 School of Medicine, National Yang Ming Chiao Tung University, Taipei, Taiwan; 5 Department of Medical Research, Taichung Veterans General Hospital, 1650 Taiwan Boulevard Sec. 4, Taichung, 40705 Taiwan; 6 Department of Food and Nutrition, Providence University, Taichung, Taiwan; 7 School of Medicine, Chung Shan Medical University, Taichung, Taiwan

**Keywords:** Allergic rhinitis, Antibiotics, Asthma, Atopic dermatitis, Prenatal exposure

## Abstract

**Purpose:**

The prevalence of atopic diseases has increased in recent decades. A possible link between antibiotic use during pregnancy and childhood atopic disease has been proposed. The aim of this study is to explore the association of antibiotic exposure during pregnancy with childhood atopic diseases from a nationwide, population-based perspective.

**Methods:**

This was a nationwide population-based cohort study. Taiwan’s National Health Insurance Research Database was the main source of data. The pairing of mothers and children was achieved by linking the NHIRD with the Taiwan Maternal and Child Health Database. This study enrolled the first-time pregnancies from 2004 to 2010. Infants of multiple delivery, preterm delivery, and death before 5 years old were excluded. All participants were followed up at least for 5 years. Antenatal antibiotics prescribed to mothers during the pregnancy period were reviewed. Children with more than two outpatient visits, or one admission, with a main diagnosis of asthma, allergic rhinitis, or atopic dermatitis were regarded as having an atopic disease.

**Results:**

A total of 900,584 children were enrolled in this study. The adjusted hazard ratios of antibiotic exposure during pregnancy to childhood atopic diseases were 1.12 for atopic dermatitis, 1.06 for asthma, and 1.08 for allergic rhinitis, all of which reached statistical significance. The trimester effect was not significant. There was a trend showing the higher the number of times a child was prenatally exposed to antibiotics, the higher the hazard ratio was for childhood atopic diseases.

**Conclusions:**

Prenatal antibiotic exposure might increase the risk of childhood atopic diseases in a dose-dependent manner.

## Introduction

With advances in medicine, antibiotics are commonly prescribed around the world [1]. The special physiology of pregnant women makes them more susceptible to infection, such as urinary tract infection. Thus, antibiotics are often used during the pregnancy period. It is estimated that up to 40% of pregnant women receive antibiotics prior to delivery [2, 3]. The prevalence of atopic diseases, such as food allergy, atopic dermatitis, asthma, and allergic rhinitis, has also increased globally in recent decades as a result of industrialization [4–6]. These allergic diseases not only seriously affect the quality of patients’ lives, but also cause a huge personal and socioeconomic burden [7].

A possible link has been suggested between the increasing use of antibiotics during pregnancy and the occurrence of atopic illnesses. The composition of an infant’s gut microbiome contributes to her subsequent immunological development. Alteration of the microbiome could lead to subsequent allergy diseases and obesity later in life [8–10]. The maternal microbiome determines the initial composition of the infant’s microbiome. Some studies reported that maternal antibiotic exposure during pregnancy could change infants’ microbiome [11, 12]. A matched case–control study found prenatal antibiotic exposure was associated with an increased risk of asthma [13]. However, large-scale studies on prenatal antibiotic exposure and atopic diseases later in life are still lacking.

The aim of this study was to explore the association of antibiotic exposure during pregnancy with childhood atopic diseases from a nationwide, population-based perspective.

## Materials and methods

### Study design and data source

This was a nationwide, population-based cohort study. Taiwan’s National Health Insurance Research Database (NHIRD) was the main source of data. Taiwan’s National Health Insurance (NHI) system was launched in 1995. It is a single-payer program with mandatory enrollment. The current coverage rate is 99.99% of Taiwan’s population (approximately 23.5 million). In 2002 , the NHIRD was established for research purposes. It contains all claims data from the NHI [14–16]. Since 2015, the Health and Welfare Data Center (HWDC) of Taiwan’s Ministry of Health and Welfare (MOHW) further integrated NHIRD with other health-related databases [17]. In this study, the pairing of mothers and children was achieved by linking the NHIRD with the Taiwan Maternal and Child Health Database (MCHD) of Taiwan’s Health Promotion Administration (HPA). The main data analyzed in this study were obtained from ambulatory care expenditures by visit (CD) files and inpatient expenditure by admission (DD) files from the NHIRD. Antibiotic exposure records were acquired from inpatient order (DO) files. For privacy protection and database reliability, Taiwan’s Ministry of Health and Welfare (MOHW) requires investigators to conduct on-site analysis. During the study period, diagnoses in the NHIRD were coded by the International Classification of Diseases, Ninth Revision, Clinical Modification (ICD-9-CM) format.

### Study population

**Fig. 1:**
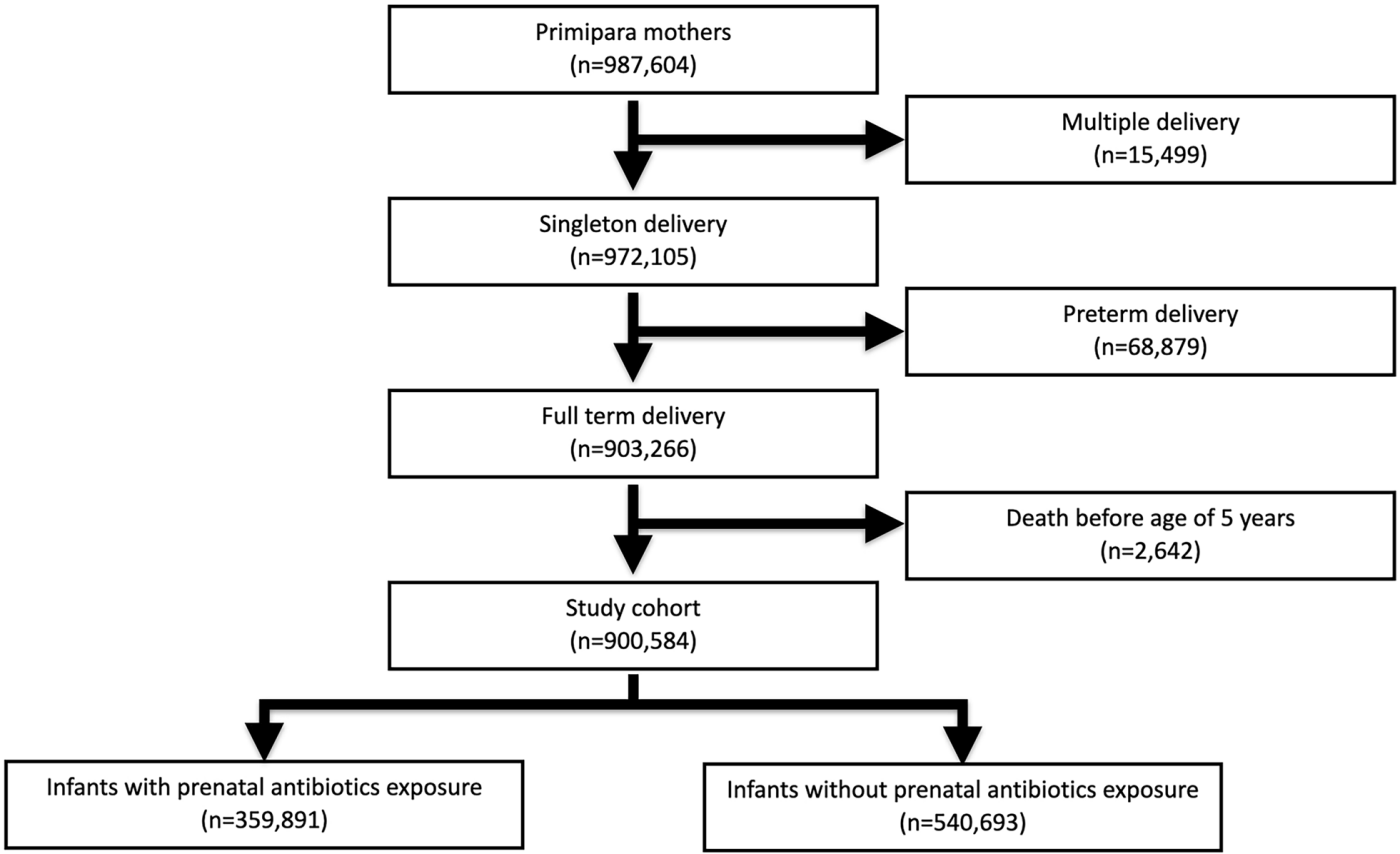
Composition of the study cohort. Only the first child in each family was enrolled. Premature infants and children of early death were excluded from analysis

This nationwide cohort study only enrolled first-time pregnancies during the study period, from 2004 to 2010. We excluded infants of multiple delivery, preterm delivery, and death before 5 years old. Finally, a total of 906,942 infants were enrolled in the study cohort (Fig. 1). The cohort was followed up until the end of 2016. All children in this cohort were followed up for at least 5 years.

### Exposure to antenatal antibiotics

Antenatal antibiotic exposure was defined as a mother who received a medication with an ATC code (J01A, J01B, J01C, J01D, J01E, J01F, J01G, J01M, J01R, and J01X) during pregnancy. The timing of prescription (first, second, and third trimesters) and cumulative numbers of prescriptions were also recorded. We restricted the subgroup analysis to the timing of the initial exposure.

### Outcome measurement

During the study period, the International Statistical Classification of Diseases and Related Health Problems, Ninth Revision, Clinical Modification (ICD9-CM) was used for coding of each diagnosis. Children who visited the outpatient department more than twice or were admitted once with a primary diagnosis of asthma (ICD-9 code 493.9), allergic rhinitis (ICD-9 code 477.9), or atopic dermatitis (ICD-9 code 691.8), were regarded as having an atopic disease.

### Covariates

Maternal age, mode of delivery, maternal comorbidities, maternal allergic diseases, pregnancy-related complications, and infants’ gender were collected as potential confounders.

### Statistical analysis

The data were retrieved and analyzed using the SAS statistical package (version 9.4; SAS Institute, Cary, North Carolina, USA). Demographic data were described by the mean with standard deviation, or frequency and percentage. Continuous variables were compared using the independent t-test. The Pearson’s Chi-square test was applied for analyzing categorical data. Cumulative incidences of atopic diseases between groups were compared by the Kaplan–Meier method. Cox regression model was applied for calculating the hazard ratios of antibiotic prescription after adjusting for potential confounders. A *p* value less than 0.05 was considered statistically significant.

## Results

### The cumulative incidences of atopic diseases

**Fig. 2:**
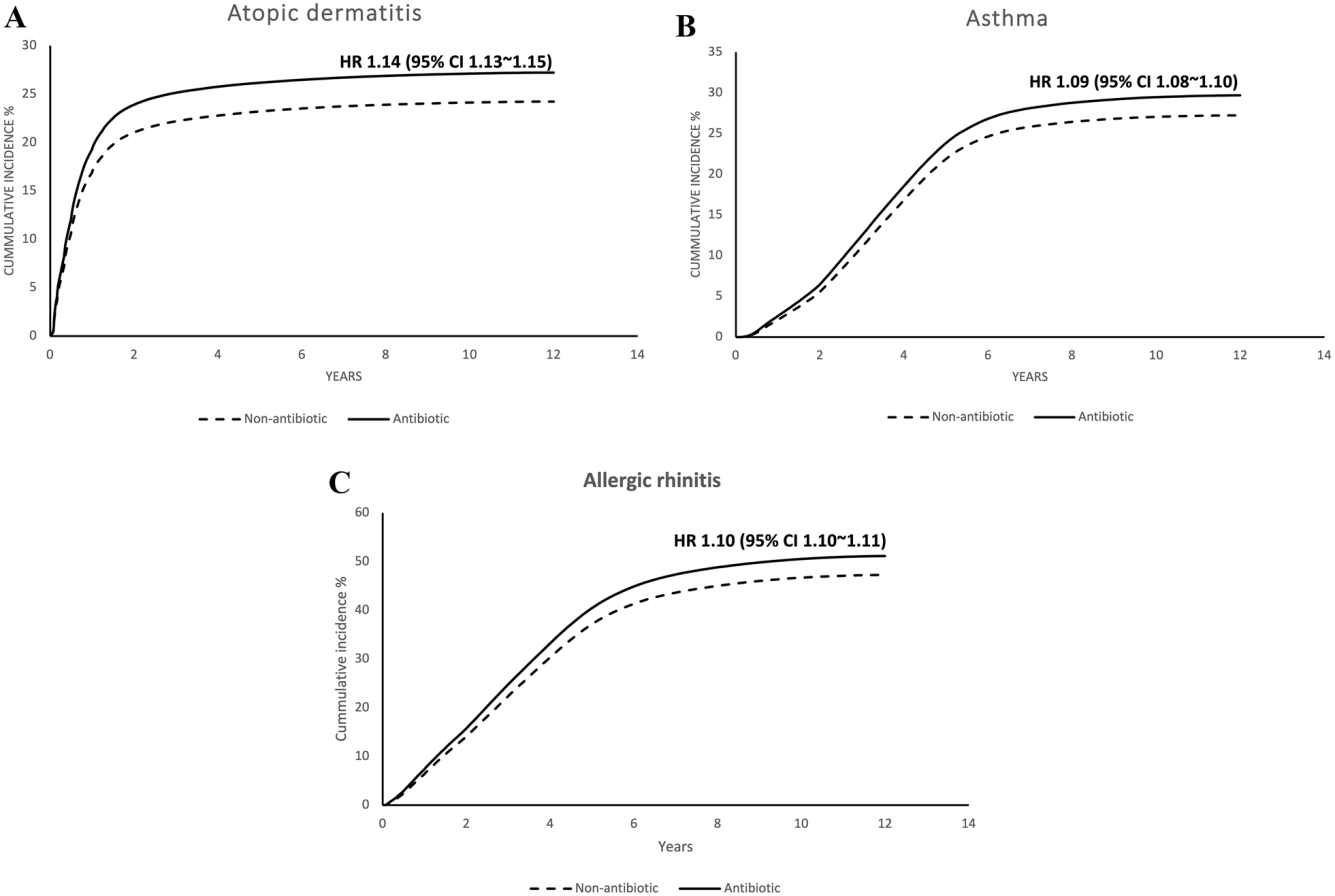
Cumulative incidences of atopic diseases with or without prenatal antibiotics: **A** atopic dermatitis; **B** asthma; **C** allergic rhinitis. Prenatal antibiotics exposure increases the cumulative risk in all three atopic diseases. *CI* confidence interval, *HR* hazard ratio

Of the 900,584 enrolled children, 359,891 (40.0%) were exposed to prenatal antibiotics. A comparison of the demographic data of these two groups revealed that the antibiotic exposure group had a slightly younger age of pregnancy, more Cesarean sections, more maternal comorbidities, more maternal allergic diseases, more pregnancy complications, and more male babies (Table 1). At the end of the study, the cumulative incidences of atopic diseases of the antibiotic exposure group were: 29.5% for atopic dermatitis, 30.5% for asthma, and 56.4% for allergic rhinitis. In the non-antibiotics group, the cumulative incidences were: 26.4% for atopic dermatitis, 28.4% for asthma, and 52.8% for allergic rhinitis (Fig. 2). The adjusted hazard ratio of antibiotics exposure during pregnancy to childhood atopic diseases were 1.12 for atopic dermatitis, 1.06 for asthma, and 1.08 for allergic rhinitis. All of them reached statistical significance (Table 2). Univariate analysis and actual numbers in each category are listed in Table 3.

**Table 1:** Characteristics of study subjects

Characteristic	Non-antibiotics group (422,740)	Antibiotics group (484,202)	Total	*p*
	*n* (%)	*n* (%)		
*Maternal age (years)*				< 0.001
< 25	72,366 (17.1)	90,395 (18.7)	162,761	
25–29	159,287 (37.7)	180,320 (37.2)	339,607	
30–34	140,761 (33.3)	151,804 (31.4)	292,565	
≥ 35	50,326 (11.9)	61,683 (12.7)	112,009	
*Mode of delivery*				< 0.001
Vaginal delivery	313,169 (74.1)	291,785 (60.3)	604,954	
Cesarean section	109,571 (25.9)	192,417 (39.7)	301,988	
*Maternal comorbidity*				
Diabetes mellitus	1822 (0.4)	3284 (0.7)	5,106	< 0.001
Hypertension	1752 (0.4)	3452 (0.7)	5,204	< 0.001
Hyperlipidemia	3365 (0.8)	6074 (1.3)	9,439	< 0.001
*Maternal allergic disease*				
Asthma	8954 (2.1)	15,594 (3.2)	24,548	< 0.001
Allergic rhinitis	65,455 (15.5)	95,671 (19.8)	161,126	< 0.001
Atopic dermatitis	7223 (1.7)	11,070 (2.3)	18,293	< 0.001
*Pregnancy-related complication*				
Anemia	15,979 (3.8)	24,690 (5.1)	40,669	< 0.001
Gestational diabetes mellitus	6217 (1.5)	7401 (1.5)	13,618	0.024
Gestational hypertension	1485 (0.4)	2389 (0.5)	3,874	< 0.001
Pre-eclampsia or eclampsia	2986 (0.7)	5390 (1.1)	8,376	< 0.001
Placenta previa or abruptio placentae	9664 (2.3)	15,581 (3.2)	25,245	< 0.001
*Neonatal gender*				< 0.001
Female	205,227 (48.5)	231,737 (47.9)	436,964	
Male	217,513 (51.5)	252,465 (52.1)	469,978	
*Timing of antibiotics exposure*				
1st trimester		288,434 (59.6)	288,434	
2nd trimester		30,173 (6.2)	30,173	
3rd trimester		165,595 (34.2)	165,595	
*Cumulative number of antibiotics*				
1 time		279,783 (57.8)	279,783	
2 times		108,969 (22.5)	108,969	
≥ 3 times		95,450 (19.7)	95,450

**Table 2:** Adjusted hazard ratios of prenatal antibiotics for childhood atopic diseases by Cox regression models*

Variables	Asthma			Allergic rhinitis			Atopic dermatitis		
	aHR	95%CI		aHR	95%CI		aHR	95%CI	
Antibiotics	1.06	1.05	1.07	1.08	1.07	1.09	1.12	1.11	1.13
Maternal age									
< 25	1.00			1.00			1.00		
25–29	1.13	1.12	1.14	1.27	1.26	1.28	1.17	1.15	1.18
30–34	1.10	1.09	1.11	1.32	1.31	1.33	1.22	1.20	1.23
≥ 35	0.97	0.96	0.99	1.21	1.20	1.23	1.10	1.09	1.12
Mode of delivery									
Cesarean section	1.05	1.04	1.05	1.02	1.02	1.03	1.05	1.04	1.06
Maternal comorbidity									
Diabetes mellitus	1.03	0.98	1.08	1.03	0.99	1.06	1.02	0.98	1.07
Hypertension	1.02	0.98	1.07	0.93	0.90	0.96	0.99	0.94	1.04
Hyperlipidemia	1.10	1.06	1.13	1.15	1.12	1.18	1.17	1.13	1.21
Maternal allergic disease									
Asthma	1.58	1.55	1.61	1.18	1.16	1.20	1.18	1.15	1.20
Allergic rhinitis	1.31	1.30	1.32	1.53	1.52	1.54	1.29	1.28	1.31
Atopic dermatitis	1.06	1.03	1.08	1.12	1.10	1.14	1.55	1.52	1.59
Pregnancy-related complication									
Anemia	1.02	0.97	1.07	0.98	0.94	1.01	0.96	0.91	1.01
Gestational diabetes mellitus	0.99	0.97	1.02	1.10	1.08	1.12	1.15	1.12	1.18
Gestational hypertension	1.06	0.95	1.18	0.97	0.90	1.06	1.02	0.91	1.14
Pre-eclampsia or eclampsia	0.91	0.84	1.00	1.00	0.94	1.06	1.00	0.91	1.09
Placenta previa and abruptio placentae	1.10	1.07	1.13	1.08	1.06	1.10	1.08	1.05	1.11
Male gender	1.33	1.32	1.34	1.29	1.29	1.30	1.04	1.03	1.05

*aHR* adjusted hazard ratio, *CI* confidence intervals

*Models adjusted for maternal age, mode of delivery, maternal comorbidity, maternal allergic disease, and pregnancy-related complications

**Table 3:** Univariate analysis of factors associated with childhood atopic diseases

Variables	Asthma					Allergic rhinitis					Atopic dermatitis				
	No	Yes	HR	95%CI		No	Yes	HR	95%CI		No	Yes	HR	95%CI	
*Antibiotics*															
No	387,072	153,621	1.00			255,452	285,241	1.00			397,702	142,991	1.00		
Yes	250,257	109,634	1.09	1.08	1.10	156,819	203,072	1.10	1.10	1.11	253,788	106,103	1.14	1.13	1.15
*Maternal age*															
< 25	118,746	44,538	1.00			85,882	77,402	1.00			123,648	39,636	1.00		
25–29	235,722	102,876	1.14	1.13	1.16	150,012	188,586	1.29	1.28	1.30	243,561	95,037	1.18	1.17	1.20
30–34	202,919	86,010	1.12	1.11	1.14	125,613	163,316	1.35	1.33	1.36	204,380	84,549	1.24	1.23	1.26
≥ 35	79,942	29,831	1.01	0.99	1.02	50,764	59,009	1.25	1.23	1.26	79,901	29,872	1.14	1.12	1.15
*Mode of delivery*															
Vaginal delivery	427,765	172,911	1.00			278,617	322,059	1.00			437,872	162,804	1.00		
Cesarean section	209,564	90,344	1.06	1.05	1.07	133,654	166,254	1.06	1.05	1.07	213,618	86,290	1.07	1.07	1.08
*Maternal comorbidity*															
Diabetes mellitus	3934	1820	1.11	1.06	1.16	2401	3353	1.14	1.10	1.18	3978	1776	1.14	1.09	1.20
Hypertension	3976	1785	1.09	1.04	1.14	2606	3155	1.03	0.99	1.07	4055	1706	1.09	1.04	1.15
Hyperlipidemia	7842	3891	1.18	1.15	1.22	4408	7325	1.28	1.26	1.31	7770	3963	1.28	1.25	1.33
*Maternal allergic disease*															
Asthma	14,592	11,887	1.80	1.77	1.83	9016	17,463	1.45	1.43	1.47	17,064	9415	1.37	1.35	1.40
Allergic rhinitis	112,758	62,428	1.38	1.37	1.39	58,829	116,357	1.58	1.57	1.59	116,308	58,878	1.35	1.34	1.37
Atopic dermatitis	13,737	6451	1.14	1.11	1.16	8099	12,089	1.22	1.19	1.24	11,928	8260	1.65	1.62	1.69
*Pregnancy-related complication*															
Anemia	3556	1507	1.04	0.99	1.09	2375	2688	0.98	0.95	1.02	3691	1372	0.98	0.93	1.03
Gestational diabetes mellitus	13,180	5417	1.01	0.98	1.03	7752	10,845	1.15	1.13	1.17	12,744	5853	1.18	1.15	1.21
Gestational hypertension	731	327	1.09	0.98	1.22	487	571	1.02	0.94	1.11	750	308	1.08	0.96	1.20
Pre-eclampsia or eclampsia	1283	502	0.96	0.88	1.05	799	986	1.04	0.97	1.10	1269	516	1.06	0.98	1.16
Placenta previa and abruptio placentae	13,930	6615	1.13	1.10	1.16	8647	11,898	1.13	1.10	1.15	14,293	6252	1.12	1.09	1.15
*Neonatal gender*															
Female	321,925	111,950	1.00			218,970	214,905	1.00			315,634	118,241	1.00		
Male	315,404	151,305	1.33	1.32	1.34	193,301	273,408	1.29	1.28	1.30	335,856	130,853	1.04	1.03	1.05

*CI* confidence intervals, *HR* hazard ratio

### Timing of prenatal antibiotic exposure and childhood atopic diseases

To investigate how the timing of antibiotic prescription affected the incidences of childhood atopic diseases, we further stratified the infants into three groups according to their first-time exposure to antibiotics during the pregnancy course. After adjusting for confounders, including maternal age, mode of delivery, preterm delivery, maternal comorbidity, maternal allergic disease, pregnancy-related complications, and neonatal gender, the hazard ratios for asthma, allergic rhinitis, and atopic dermatitis were 1.07, 1.09, and 1.13 for the first trimester, 1.06, 1.06, and 1.07 for the second trimester, and 1.02, 1.04, and 1.06 for the third trimester. Although all these hazard ratios reached statistical significance, the timing of exposure did not affect the magnitude of risk for childhood atopic diseases (Table 4).

**Table 4:** Adjusted hazard ratios of prenatal antibiotics for childhood atopic diseases by Cox regression models*

	Asthma		Allergic rhinitis	Atopic dermatitis		
	HR	95%CI	HR	95%CI	HR	95%CI
*Stratified by timing of prescribing antibiotics*						
1st trimester	1.07	1.06 ~ 1.08	1.09	1.08 ~ 1.09	1.13	1.12 ~ 1.14
2nd trimester	1.06	1.03 ~ 1.08	1.06	1.05 ~ 1.08	1.07	1.04 ~ 1.09
3rd trimester	1.02	0.99 ~ 1.04	1.04	1.02 ~ 1.06	1.06	1.04 ~ 1.09
*Stratified by cumulative times of antibiotics prescription*						
1 time	1.04	1.04 ~ 1.05	1.06	1.06 ~ 1.07	1.08	1.07 ~ 1.09
2 times	1.06	1.05 ~ 1.08	1.09	1.08 ~ 1.10	1.13	1.11 ~ 1.14
≥ 3 times	1.11	1.09 ~ 1.12	1.12	1.11 ~ 1.13	1.20	1.19 ~ 1.22

*Model adjusted for maternal age, mode of delivery, preterm delivery, maternal comorbidity, maternal allergic disease, pregnancy-related complication, neonatal gender; *CI* confidence interval

### Cumulative number of times of prenatal antibiotic exposure and childhood atopic diseases

We further stratified the children according to their cumulative number of times of prenatal antibiotics exposure to test if a dose-dependent effect existed. After adjusting for confounders, including maternal age, mode of delivery, preterm delivery, maternal comorbidity, maternal allergic disease, pregnancy-related complication, and neonatal gender, the hazard ratios for asthma, allergic rhinitis, and atopic dermatitis were 1.04, 1.06, 1.08 for one exposure, 1.06, 1.09, 1.13 for two exposures, and 1.11, 1.12, 1.20 for exposure more than 3 times. A trend was revealed showing the higher the number of times an infant was prenatally exposed to antibiotics, the higher the hazard ratio was for childhood atopic diseases (Table 4).

### Types of delivery and risk for childhood atopic diseases

We stratified the children according to their types of delivery. After adjusting for potential confounders, including maternal age, mode of delivery, preterm delivery, maternal comorbidity, maternal allergic disease, pregnancy-related complication, and neonatal gender, the hazard ratios for asthma, allergic rhinitis, and atopic dermatitis were 1.07, 1.08, 1.12 for vaginal delivery and 1.06, 1.08, 1.12 for Cesarean section (Table 5). The risk raised by antibiotics exposure was not modified by types of delivery.

**Table 5:** Adjusted hazard ratios of prenatal antibiotics for childhood atopic diseases, stratified by types of delivery*

Variables	Asthma			Allergic rhinitis			Atopic dermatitis		
	HR	95%CI		HR	95%CI		HR	95%CI	
*Types of delivery*									
Vaginal delivery	1.07	1.06	1.08	1.08	1.08	1.09	1.12	1.11	1.13
Cesarean section	1.06	1.04	1.07	1.08	1.07	1.09	1.12	1.11	1.14

*Model adjusted for maternal age, mode of delivery, preterm delivery, maternal comorbidity, maternal allergic disease, pregnancy-related complication, neonatal gender

## Discussion

This nationwide, population-based cohort study reveals that prenatal antibiotic exposure increases the risk of childhood atopic disease. A dose-dependent effect was revealed by the positive correlation between the cumulative number of times antibiotics were prescribed and the risk of atopic diseases. The increased risk of atopy associated with antibiotic exposure was not affected by different trimesters. This study provides comprehensive evidence that the pathogenesis of childhood allergic diseases may begin in early pregnancy, according to population-based data.

Antibiotic exposure in mid-to-late pregnancy was consistently associated with childhood asthma in a Danish birth cohort study [18]. Trimester effects have also been reported in several smaller-scale studies [19–21]. However, a meta-analysis revealed a positive association between prenatal antibiotic use in every trimester and the occurrence of childhood asthma [22]. In our study, trimester effects were assessed, but no significant differences in hazard ratios were found among the trimesters. This apparent inconsistency in findings might be explained by the different follow-up periods and different disease definitions used in the studies. Prenatal antibiotic exposure has also been reported to increase the risk of atopic dermatitis and hay fever [23, 24]. However, the trimester effects were not analyzed. Our study also reported that risk of allergic rhinitis was positively associated with prenatal antibiotic exposure. Respiratory tract infections were the most common indication for prenatal antibiotics use in our study (Appendix Table 6). Subgroup analysis by different kinds of antibiotics is added in Appendix Table 7. There are no significant differences between groups. Only quinolone shown borderline statistical significance in asthma. Vaginal delivery exposes the newborn to the maternal gut microbiota directly during birth, which may have a protect effect than in cases of cesarean section.

More studies are needed to elucidate the mechanism underlying the positive association between use of prenatal antibiotics and childhood atopic diseases. The hygiene hypothesis may partially explain it [25]. According to the hygiene hypothesis the microbiota, i.e., the composition of the intestinal flora, which is established early in life, plays a crucial role in the development of the immune system in children [26]. The association between gut microbiota and allergic diseases has been reported in a number of studies [27, 28]. The microbial colonization of the fetus has been reported to occur as early as 11 weeks of gestation [29]. Thus, by inducing reductions and alterations in the fetal intestinal microbiota, exposure to antibiotics during pregnancy may affect immune system development, thereby increasing the likelihood of chronic disease [30, 31]. Animal studies have also shown that antibiotics could induce the transition from TH_1_/TH_2_ balance to TH_2_-dominant immunity. Nevertheless, oral administration of intestinal flora could prevent this process from developing [32, 33]. The risk of childhood asthma increases as the cumulative number of courses of prenatal antibiotics increases, according to a Canadian cohort study [34]. A dose-dependent effect has also been reported in a claims data analysis [31]. In our study, a similar trend was noted in all childhood allergic diseases. This further supports the notion that prenatal antibiotics may be causally linked with childhood atopic diseases, and that this relationship is not the result of the phenomenon of confounding by indication [35, 36].

The correlation between antibiotic exposure during pregnancy and childhood allergic diseases may be confounded by many factors. Maternal characteristics such as maternal age, maternal history of allergy, maternal smoking, delivery mode, and maternal education level have all been reported [23, 34, 35, 37, 38]. The strongest confounder may be maternal allergic disease, because atopy has a strong hereditary tendency. The strongest predictor of childhood atopic diseases is genetic inheritance from parents. If we include all siblings in this study. The analysis might be confounded by family clusters [39, 40] So, we included only the first child in each family. Preterm infants usually have more medical care need. So, we excluded them to prevent the confounding effect. If children did not survive more than 5 years, short follow-up time would confound the outcome analysis. As a result, we did not involve those infants of early death.

Our study had certain limitations. The data source was national health insurance claims data, which do not include laboratory data. The disease diagnosis was mainly decided by physicians’ coding. The validity of the diagnoses could not be confirmed because personal identification data are not permitted to be released from the data center. Thus, certain misclassifications may have existed. Because we used Cox regression model to analyze the cumulative hazard ratio between groups. However, Cox regression model (proportional hazard model) can only calculate the hazard ratio. Risk difference calculation can count the attributable risk proportion. It may be more valuable in public health policy making.

## Data Availability

In this study, the data analyzed are subject to the following licenses/restrictions: To protect patients’ identity and validate the reliability of the databases, investigators are required to perform onsite analysis at HWDC via remote connection to MOHW servers. Requests to access these datasets should be directed to Dr. Ching-Heng Lin, epid@vghtc.gov.tw.

## Appendix

See Tables 6 and 7.

**Table 6:** The top 20 indication for prenatal antibiotics

ICD9	Diagnosis	*n*	Percent	Cumulative numbers	Cumulative percent
465	Acute pharyngitis	46,281	12.86	46,281	12.86
463	Tonsilitis	24,645	6.85	70,926	19.71
461	Sinusitis	22,190	6.17	93,116	25.88
616	Inflammatory disease of cervix, vagina, and vulva	20,439	5.68	113,555	31.56
599	UTI	18,369	5.11	131,924	36.67
V22	Normal pregnancy	16,239	4.51	148,163	41.18
595	Cystitis	15,738	4.37	163,901	45.55
789	Abdominal pain	13,140	3.65	177,041	49.21
614	Inflammatory disease of ovary, fallopian tube, pelvic cellular tissue, and peritoneum	10,756	2.99	187,797	52.19
523	Gingival and periodontal diseases	8659	2.41	196,456	54.6
626	Disorders of menstruation and other abnormal bleeding from female	8168	2.27	204,624	56.87
466	Acute bronchitis and bronchiolitis	7897	2.19	212,521	59.07
640	Hemorrhage in early pregnancy	7663	2.13	220,184	61.2
706	Diseases of sebaceous glands	6862	1.91	227,046	63.1
462	Pharyngitis, acute	6282	1.75	233,328	64.85
644	Early or threatened labor	6033	1.68	239,361	66.53
487	Influenza	5406	1.5	244,767	68.03
646	Other complications of pregnancy, not elsewhere classified	4905	1.36	249,672	69.39
460	Acute nasopharyngitis (common cold)	4894	1.36	254,566	70.75
558	Other noninfectious gastroenteritis and colitis	4764	1.32	259,330	72.08

**Table 7:** Subgroup analysis by different kinds of antibiotics

Variables	Asthma			Allergic rhinitis			Atopic dermatitis		
	HR	95%CI		HR	95%CI		HR	95%CI	
*Type of antibiotics*									
J01A (tetracyclines)	1.09	1.06	1.12	1.13	1.11	1.15	1.19	1.16	1.22
J01B (amphenicols)	1.09	1.04	1.15	1.08	1.04	1.12	1.08	1.03	1.14
J01C (beta-lactam antibacterials, penicillins)	1.08	1.07	1.10	1.08	1.07	1.09	1.11	1.10	1.12
J01D (other beta-lactam antibiotics)	1.04	1.03	1.05	1.08	1.07	1.09	1.12	1.11	1.13
J01E (sulfonamides and trimethoprim)	1.08	1.04	1.12	1.13	1.10	1.16	1.10	1.05	1.14
J01F (macrolides, lincosamides and streptogramins)	1.06	1.05	1.08	1.09	1.08	1.11	1.13	1.11	1.15
J01G (aminoglucosides)	1.12	1.05	1.20	0.98	0.93	1.04	1.02	0.95	1.10
J01M (quinolone)	1.02	0.99	1.06	1.07	1.04	1.10	1.13	1.09	1.17
J01R (combination)									
J01X (other antibacterials)	1.05	1.00	1.10	1.08	1.04	1.12	1.11	1.06	1.17

Model adjusted for maternal age, mode of delivery, preterm delivery, maternal comorbidity, maternal allergic disease, pregnancy-related complication, neonatal gender

## Abbreviations

HPA: Health Promotion Administration
HWDC: Health and Welfare Data Center
ICD-9-CM: International Classification of Diseases, Ninth Revision, Clinical Modification
MCHD: Maternal and Child Health Database
MOHW: Ministry of Health and Welfare
NHI: National Health Insurance
NHIRD: National Health Insurance Research Database
